# A human tissue-based functional assay platform to evaluate the immune function impact of small molecule inhibitors that target the immune system

**DOI:** 10.1371/journal.pone.0180870

**Published:** 2017-07-18

**Authors:** Cristina St. Pierre, Jane Guo, John D. Shin, Laura W. Engstrom, Hyun-Hee Lee, Alan Herbert, Laura Surdi, James Baker, Michael Salmon, Sanjiv Shah, J. Michael Ellis, Hani Houshyar, Michael A. Crackower, Melanie A. Kleinschek, Dallas C. Jones, Alexandra Hicks, Dennis M. Zaller, Stephen E. Alves, Ravisankar A. Ramadas

**Affiliations:** 1 Oncology & Immunology Discovery, Merck Research Laboratories, Boston, MA, United States of America; 2 Genetics and Pharmacogenomics, Merck Research Laboratories, Boston, MA, United States of America; 3 Drug Metabolism and Pharmacokinetics, Merck Research Laboratories, Boston, MA, United States of America; 4 Pharmacology, Merck Research Laboratories, Boston, MA, United States of America; 5 Exploratory Chemistry, Merck Research Laboratories, Boston, MA, United States of America; Istituto di Genetica Molecolare, ITALY

## Abstract

While the immune system is essential for the maintenance of the homeostasis, health and survival of humans, aberrant immune responses can lead to chronic inflammatory and autoimmune disorders. Pharmacological modulation of drug targets in the immune system to ameliorate disease also carry a risk of immunosuppression that could lead to adverse outcomes. Therefore, it is important to understand the ‘immune fingerprint’ of novel therapeutics as they relate to current and, clinically used immunological therapies to better understand their potential therapeutic benefit as well as immunosuppressive ability that might lead to adverse events such as infection risks and cancer. Since the mechanistic investigation of pharmacological modulators in a drug discovery setting is largely compound- and mechanism-centric but not comprehensive in terms of immune system impact, we developed a human tissue based functional assay platform to evaluate the impact of pharmacological modulators on a range of innate and adaptive immune functions. Here, we demonstrate that it is possible to generate a qualitative and quantitative immune system impact of pharmacological modulators, which might help better understand and predict the benefit-risk profiles of these compounds in the treatment of immune disorders.

## Introduction

A normally functioning immune system is key for the health and survival of humans, while aberrant immune responses lead to the development of a plethora of chronic inflammatory and autoimmune disorders [1, 2]. While the same cellular and molecular components of the immune system are responsible for both protective and detrimental outcomes, the nature of the outcome is defined by the context, quality, magnitude and duration of the immune response.

Pharmacological modulation of targets and pathways in the immune system has been successful in providing clinical benefit in a variety of inflammatory and autoimmune diseases such as asthma, rheumatoid arthritis, systemic lupus erythematosus and inflammatory bowel disease [3]. While several pharmacological modulators have a well-characterized direct mechanism of action (MoA) based on their molecular targets, others have less-characterized, indirect or multiple MoAs. For example, corticosteroids exert their anti-inflammatory effects by general modulation of transcriptional responses in target cells leading to a broader immune impact [4]. The recently approved Janus Kinase (JAK) inhibitors target the JAK-STAT pathway, leading to a narrower spectrum of cytokine mediated immune impact [5]. On the other hand, selective antagonism of histamine binding to the histamine H_1_ receptor leads to a focused biological impact by preventing the release of inflammatory mediators from mast cells and basophils and providing therapeutic benefit in allergic diseases [6]. Depending on the stage of the drug discovery process, pharmacological modulators are evaluated in assays aimed at assessing the properties of the compound and pathway investigated that most pertains to the proposed MoA of the drug target [7]. These assays are usually compound- and mechanism- centric and might not reflect the *in vivo* impact of this compound. Therefore, a comprehensive, systems biology and systems pharmacology approach has been proposed as a plausible path forward to better understand and predict the impact of compounds and drugs *in vivo* [8–10].

The individual human responses to a drug can vary widely, depending on many factors such as disease heterogeneity, environmental factors as well as genetics [11]. Since treatment of chronic inflammatory and autoimmune disorders require a chronic dosing paradigm, the success or failure of a drug depends on the benefit-risk ratio evaluated in the context of efficacy and safety. The drug response and the benefit-risk profile of a given drug are heterogeneous in a clinical setting [12]. While both biologics and small molecule drugs are used for the treatment of chronic inflammatory and autoimmune diseases, small molecule drugs especially can have a broader impact by having an effect on more than one pathway, especially in target classes such as kinases [13, 14]. Therefore, the concern of general immunosuppression is a clinical concern and an area of active preclinical research. Often, there are limited efforts towards a streamlined and consistent interrogation of the impact of these compounds across all the key components of the immune system. Even when such efforts are undertaken, the scope of the studies and the range of available data are either limited or dispersed across studies in published literature. Since perturbations in the immune system invariably impacts most of the organ systems in humans, a consistent evaluation of the broad immune function impact of pharmacological modulators might provide a better understanding of the organ system impact in the context of efficacy, safety and adverse events in drug discovery and development.

Therefore, we established a panel of immune function assays using human peripheral blood mononuclear cells (PBMC) or whole blood to develop an ‘immune fingerprint’ for small molecule drugs. We used this assay platform to systematically evaluate the immune function impact of standard of care (SoC) compounds used as therapeutics in the clinical setting, as well as small molecule inhibitors against several intracellular targets in the immune system. Our data demonstrate that the characterization of a comprehensive immune function impact profile of a pharmacological inhibitor may help better understand and predict the benefit-risk profiles of these compounds in a translational setting.

## Materials and methods

### Compounds used in the study

Methotrexate, prednisolone, leflunomide, mycophenolate mofetil, rapamycin, cyclosporine A, BAY 11–7082, latrunculin A and diphenyliodonium chloride (DPI) were purchased from Sigma Aldrich (St. Louis, MO). TLR agonists and BAY-11 were purchased from Invivogen (San Diego, CA). Small molecule inhibitors targeting Janus kinase (JAK), spleen tyrosine kinase/zeta-chain associated protein kinase 70 (SYK/ZAP-70), bruton tyrosine kinase (BTK), Interleukin-1 receptor associated kinase 4 (IRAK4) and Phosphoinositide 3 kinase delta (PI3Kδ) were synthesized at Merck & Co., Inc., Kenilworth, NJ, USA. These compounds were potent at nanomolar concentrations in target-specific *in vitro* biochemical assays, and contained varying levels of selectivity against related enzymes in the pathway. All compounds were reconstituted in dimethyl sulfoxide (DMSO) and dispensed in the assay plates either by manual pipetting or using Echo automated liquid handler (Labcyte, Sunnyvale, CA).

### Human tissue samples

Frozen stocks of normal human PBMCs were obtained from Precision BioServices (Frederick, MD). Cryopreserved samples were thawed in a 37°C water bath, added to prewarmed culture media, centrifuged at 300g for 5 minutes, and resuspended to desired concentrations. Unless otherwise specified, the culture medium used for all the PBMC assays contained RPMI supplemented with 5% fetal bovine serum, 50U/mL penicillin, and 50μg/mL streptomycin. Fresh human blood samples were collected in sodium heparin tubes from normal healthy donors who provided their written informed consent and were enrolled in the WIRB–compliant Merck Research Blood Donor Program at Merck & Co., Inc, Boston, MA, USA.

### Assay to evaluate T cell activation and proliferation

Peripheral blood mononuclear cells in culture media were pre-treated with compounds (0.1% final DMSO concentration) for 45 minutes at 37°C, 5% CO_2_ and then transferred into anti-CD3 (1 μg/mL, OKT3, eBioscience, San Diego, CA) coated plate containing soluble anti-CD28 (1 μg/mL, Fitzgerald, Acton, MA). Twenty four hours after stimulation, an aliquot of cell culture supernatant was collected and evaluated for IL-2 production (MesoScale Discovery, Rockville, MD) according to manufacturer’s instructions. The remaining culture was allowed to continue incubation for an additional 48 hours. To evaluate proliferation, Click-iT EdU Alexa Flour 647 Assay Kit (Invitrogen, Carlsbad, CA) was used according to manufacturer’s instructions, and proliferation was evaluated at 72 hours following stimulation. The cells were stained with flourochrome conjugated antibodies against CD3, CD4 and CD8 and analyzed in a BD Fortessa flow cytometer by acquiring 8–10,000 events. The data was analyzed by FACSDiva or FlowJo and reported as the percentage of EdU^+^CD3^+^CD4^+^ and EdU^+^CD3^+^CD8^+^ lymphocytes (Figure A in S2 Fig).

### Assay to evaluate NK cell killing function

A commercially available kit was used to asses NK cell killing (NK Test, Glycotope Biotechnology). Human PBMCs (effector cells) resuspended in culture medium were plated in a round bottom, sterile poly propylene 96 well plate (Corning #3359) and incubated with compounds (final DMSO concentration 0.1%) for 1 hour. Membrane labeled GFP positive K562 cells (target cells) were then added to the effector cells at a 1:50 target–effector ratio. Cells were centrifuged for 3 minutes at 120xg to ensure cell-cell contact and incubated at 37°C, 5% CO2 for 2 hours. Cells were then resuspended in propidium iodide staining solution and incubated at room temperature for 5 minutes. A total of 10,000 target cell events were acquired on a BD Fortessa flow cytometer, and the data was analyzed by FACSDiva or Flowjo. Percentage of GFP^+^ PI^+^ cells were reported as target cells killed under assay conditions (Figure B in S2 Fig).

### Assay to evaluate phagocyte ROS production

Phagocytic oxidative burst was assessed in whole blood samples using the Phagoburst kit (Orpegen Biopharma, #10–0200). The assay was modified to accommodate a 96 well format. Briefly, 50 μL human whole blood was mixed well with compounds (0.1% final DMSO concentration) and incubated for 30 minutes at 37°C, 5% CO2. Then, samples were incubated on ice for 5 minutes, followed by the addition of 10uL of reagent B (opsonized bacteria). Samples were then mixed well and incubated for 10 minutes in a 37°C water bath. Next, 10μL of reagent E (substrate solution) was added to the samples, mixed well and incubated for an additional 10 minutes in the 37°C water bath. Then, 150uL of reagent F (lysis solution) was added to all wells, mixed well and incubated for 20 minutes at room temperature. Samples were washed twice with reagent A and incubated with 90μL of reagent G (DNA staining solution) for 10 minutes, on ice and protected from light. The samples were analyzed in a BD Fortessa flow cytometer. Cells were gated based on Forward and Side scatter properties and high content DNA. A total of 10,000 cell events (high content DNA) were collected, analyzed using FACSDiva or Flowjo and the oxidative burst was measured by the geometric mean fluorescence intensity (GMFI) of the ROS detection dye in the assay (Figure C in S2 Fig).

### Assay to evaluate TLR responses

To evaluate TLR3 responses, frozen PBMCs were thawed in culture medium. A total of 2x10^5^ PBMCs were seeded per well in a 96-well tissue culture plate for the assay. One hour following incubation with compounds at 37°C, 5% CO_2_, the PBMCs were treated with a PBMC donor-specific EC_80_ concentration of the high molecular weight Poly I:C (TLR3 ligand, Invivogen, San Diego, CA) or appropriate controls, and were allowed to incubate at 37°C, 5% CO_2_ for 24 hours. Twenty four hours following incubation, 120μl of the cell culture supernatant was removed from the cell culture plates and frozen at -80°C until further analysis. To evaluate TLR7 and TLR9 responses, PBMCs were thawed in culture medium, reconstituted to a concentration of 3x10^6^ cells/ml, dispensed into ultra-low attachment flasks and incubated at 37°C, 5% CO_2_ for 24h with continuous rocking. The PBMCs were harvested from the flask, seeded at a density of 2x10^5^ PBMCs/well in compound-dispensed assay plates and allowed to incubate at 37°C, 5% CO_2_ for 24 hours. One hour following incubation with compounds at 37°C, 5% CO_2_, the PBMCs were treated with a PBMC donor-specific EC_80_ concentration of high molecular weight CL264 (TL7 ligand, Invivogen, San Diego, CA) or ODN 2395 (TLR9 ligand, Invivogen, San Diego, CA), or appropriate controls, and were allowed to incubate at 37°C, 5% CO_2_ for 24 hours. Twenty four hours following incubation, 120μl of the cell culture supernatant was removed from the cell culture plates and frozen at -80°C until further analysis. The cell culture supernatants were analyzed for IP-10 or IL-6 levels using MSD kits (Meso Scale Discovery, Rockville, MD) according to manufacturer’s protocols.

### Nanostring mRNA profiling and analysis

Cell pellets from experiments were lysed in RLT buffer and prepared according to standard protocol recommended by NanoString Technologies. Briefly, lysates were co-incubated with capture and reporter probes (NanoString Technologies Immunology v2 Codeset) in a thermal cycler set at 65°C for 24 hours. Samples were processed and analyzed with a Generation 2 nCounter. Counts from samples were normalized using housekeeping genes scored on the Nanostring platform. Samples with counts less than 20 were excluded from further analysis. The Matlab function ttest2 was used to determine p-values for log2 fold changes, and the function agglomerative was used to perform clustering.

### Plasma protein and media binding

Fresh plasma from EDTA-treated blood from humans was purchased from Bioreclamation IVT (Westbury, NY). The plasma protein binding of test compounds was determined using equilibrium dialysis with 96-well dialyzer plates produced by HTD dialysis LLC (Gales Ferry, CT). Compounds were spiked at 1 μM in plasma. The dialysis chambers for plasma and buffer (100 mM phosphate buffer, pH7.4) were separated by a semipermeable membrane (12–14 kDa cutoff). After 6 hours of incubation on a single-plate rotator at 37°C inside a CO_2_ chamber maintained at 5% CO_2_, matrix-matched aliquots of plasma and buffer were mixed with acetonitrile containing generic internal standard mixture. Following centrifugation, samples were analyzed using LC-MS/MS. The fraction unbound in plasma (fu,p) was calculated as follows: fu,p = (peak area ratio in buffer)/(peak area ratio in plasma). A similar methodology was followed for media binding. In this case, test compound was spiked at 1 μM into media plus 5% fetal bovine serum (FBS). The HTD dialysis chamber on the other side of the semipermeable membrane contained media only. The fraction unbound in media (fu,media) was calculated as follows: fu,media = (peak area ratio in media)/(peak area ratio in media plus 5% FBS).

### Assay principles used

The stimulant was the chemical agent that was used to elicit the response in the assay (i.e., TLR ligands in the TLR assay). We used EC_80_ stimulation conditions for evaluating the impact of compounds in assays with titrable stimulants. To account for donor-to-donor variability in PBMCs, we used donors that whose EC_80_ values were within a 3-fold range determined by dose-response experiments with the stimulants. For the NK cell killing assay, we used the conditions and donors that gave the best assay window (i.e., 1:50 target:effector ratio). For the T cell stimulation assay, we performed a dose-titration of αCD3 and αCD28 to determine the stimulation conditions used in this assay. For the phagocyte oxidative burst assay, since we could not screen human whole blood for selecting best responders, we followed the manufacturer’s protocol on 8–10 different human whole blood samples. Every assay plate contained samples that would elicit a minimal response (no stimulant), maximal response (stimulant with no compounds), compound dose-response (stimulant with compounds) and positive control (stimulant with compound that is known to inhibit the assay). We evaluated the impact of small molecule inhibitors in 8–10 different doses for every PBMC sample in every assay. The values obtained from multiple PBMC donors were compiled together to calculate the inhibitory concentrations of the compounds in a given assay.

### Data analysis and statistics

Each assay contained samples that would elicit a minimal and a maximal response, which defined the assay window. The data obtained from compound dose-response experiment were normalized to the maximal and minimal response to obtain the percentage of inhibition and analyzed using the nonlinear regression (curve fit) function in Graphpad Prism (Graphpad Software, La Jolla, CA) to calculate inhibitory concentrations. The ‘top’ parameter of the ‘best fit values’ in the analysis was used to denote the % maximal inhibitory activity achieved in the assay. The IC_50_ values obtained from the original dose-response curves were corrected for plasma protein binding using the calculations described in the ‘*Plasma protein and media binding*’ section in the methods, and the plasma protein binding-corrected inhibitory concentrations are reported for all the compounds, except assay controls such as latrunculin, BAY-11 and DPI.

## Results

### Assays to evaluate immune function impact of small molecule inhibitors

**Fig 1:**
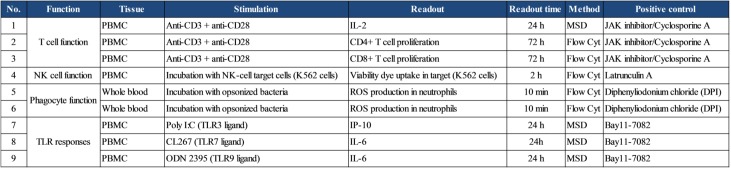
Assays to evaluate immune function impact of small molecule inhibitors. All the assays were established using healthy donor peripheral blood mononuclear cells (PBMCs) or whole blood. The immune functions evaluated, the tissues analyzed, stimulations used as well as the tissue analysis methods are listed for each assay. Small molecule inhibitors that are known to inhibit these functions were used as positive controls (assay controls) in each run of the assay, to ensure consistent technical performance and data robustness.

A panel of immune function assays that address T cell activation and proliferation, NK cell killing activity, phagocyte responses and TLR responses were established using healthy human PBMCs or whole blood. Since the *in vivo* human immune response is a result of complex interplay between multiple cell types, we decided to use human PBMCs or whole blood to develop the assays instead of using purified immune cell populations. Details of tissues used, type of readouts evaluated in the individual assays, the time of readouts, the methods used for analyses as well as assay-specific positive control inhibitors are listed in Fig 1.

### Immune function impact profiles of small molecule inhibitors

**Fig 2:**
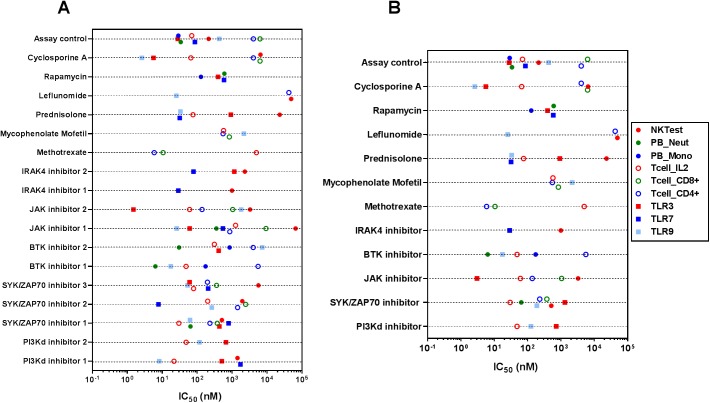
Immune function impact profiles of small molecule inhibitors. Small molecule inhibitors were evaluated in six different functional assays with nine different read-outs. For a given compound on the Y-axis, each symbol on the X-axis represents the protein binding corrected cellular potency of that compound in the corresponding assay. The potencies of the evaluated compounds in these assays are displayed as IC_50_ values in the X-axis. The reported IC_50_ values were generated from a composite of 8–10 point dose response curves from n = 6–8 donors for each compound in each assay. Immune function impact of a broader set (A) or a subset (B) of inhibitors are presented.

We evaluated the dose-dependent inhibition of immune function by small molecule inhibitors to generate a qualitative comparison of the overall ‘immune impact profile’ of the small molecule inhibitors evaluated (Fig 2A). We used compound-specific half-maximal inhibition of the response (IC_50_) in the assays and percentage of maximal inhibition achieved by these compounds in the assays (S1 Fig) to facilitate a qualitative comparison of the immune function impact of the evaluated inhibitors. Assay controls, the compounds that have been shown to exert an inhibitory effect in that specific assay, were potent in near-maximally inhibiting the respective activities. Such datasets, generated with the compounds in six separate functional assays with nine distinct functional readouts, provide an overview of the diversity of the immune function impact of compounds with known immunosuppressive properties. For example, cyclosporine has inhibitory activities on six of the nine tested immune functions, while rapamycin, prednisolone and methotrexate had inhibitory effects on four, five and three of the tested immune functions (Fig 2A). It is also discernible from this data that are qualitative (number and type of immune functions impacted) and quantitative (potency) differences between compounds developed against the same drug target. While most of the evaluated compounds had enzymatic assay potencies in the low nanomolar range, the IC_50_ values in these cell-based functional assays ranged from low nanomolar to high micromolar concentrations in the concentration ranges evaluated in this assay platform (Fig 2A).

### The effect of small molecule inhibitors on human T cell function

**Fig 3:**
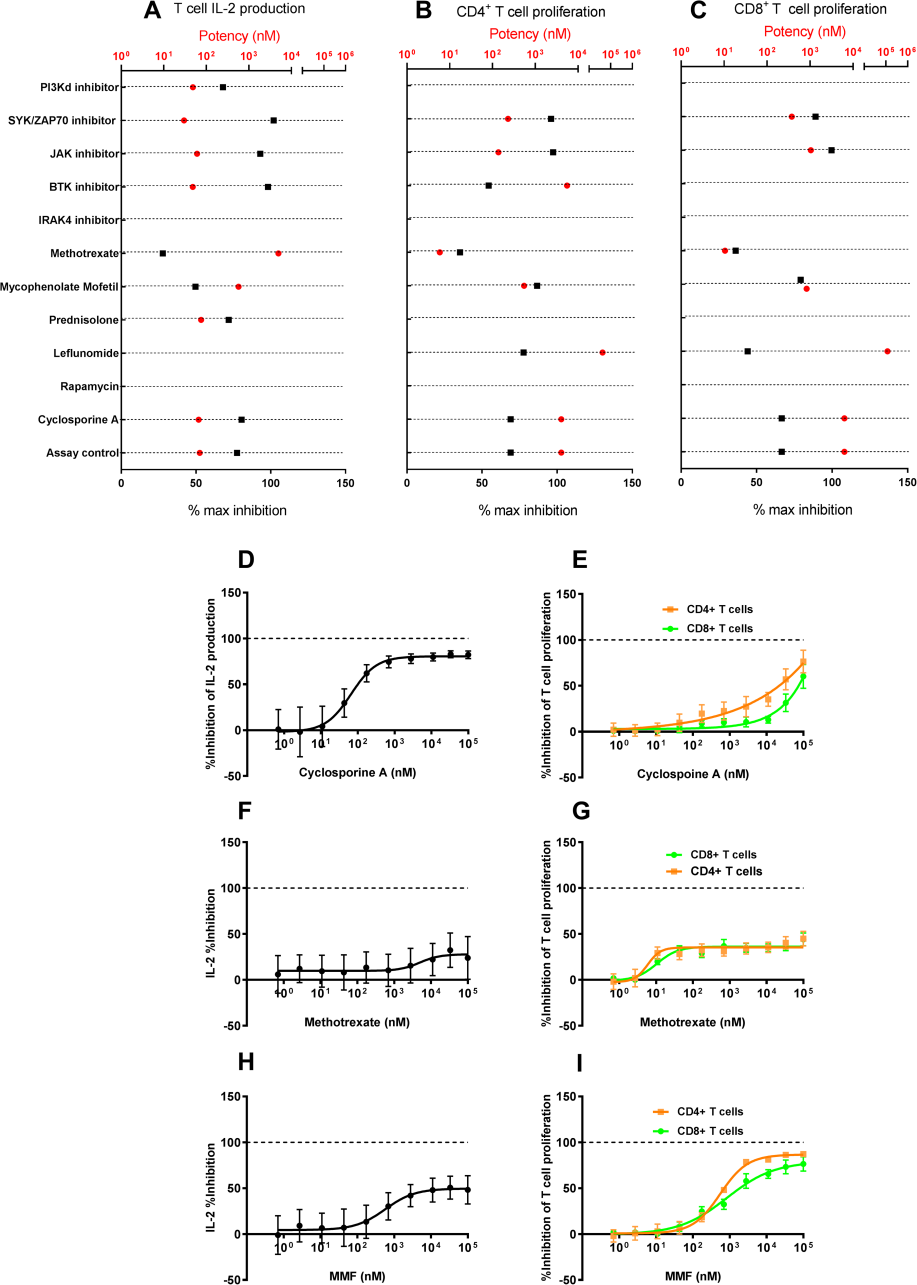
The impact of small molecule inhibitors on human T cell function. The production of IL-2 as well as the proliferation of CD4^+^ and CD8^+^ T cells was evaluated in a human *in vitro* PBMC assay. The production of IL-2 was evaluated 24h following stimulation with α-CD3 and α-CD28 while the proliferation of CD4^+^ and CD8^+^ T cells was evaluated in the same cultures 72h following α-CD3 and α-CD28 stimulation. Small molecule inhibitors suppress α-CD3 and α-CD28 induced IL-2 production (A), CD4^+^ T cell proliferation (B) and CD8^+^ T cell proliferation (C) in human PBMCs. For each compound, the potency (red circles) is plotted along the top X-axis and the percentage maximal inhibition achieved in the assay (black squares) are plotted along the bottom X-axis. The potencies of the evaluated compounds in these assays are displayed as IC_50_ ovalues. A maximum of 100% inhibition is possible for small molecule inhibitors in these assays. The reported potency and % max inhibition values were generated from a composite of 8–10 point dose response curves from n = 6–8 donors for each compound.

When we evaluated the effect of SM inhibitors in human T cell activation assays, the T cell immune impact profile we obtained for many compounds agreed with previous reports, but we also uncovered some unexpected findings (Fig 3A–3C). For example, the PI3Kδ inhibitor we evaluated in this assay only inhibited IL-2 production (Fig 3A), but did not have any significant impact on either CD4^+^ (Fig 3B) or CD8^+^ (Fig 3C) T cell proliferation. On the other hand, a SYK/ZAP70 inhibitor impacted IL-2 production as well as CD4^+^ and CD8^+^ T cell proliferation. We also observed that a BTK inhibitor we evaluated in this assay also had an impact on IL-2 production, at an IC_50_ of 48 nM, with a near complete inhibition of IL-2 production. The JAK inhibitors evaluated in the assay inhibited both IL-2 production as well as CD4^+^ and CD8^+^ T cell proliferation. Small molecule inhibitors of IRAK4 did not have an impact on IL-2 production and T cell proliferation. Among the other compounds tested, cyclosporine inhibited IL-2 production (Fig 3D) but did not demonstrate a sigmoidal dose-dependent inhibition of T cell proliferation (Fig 3E). Similarly, prednisolone inhibited only IL-2 production but did not have an impact on T cell proliferation. Methotrexate had a marginal but variable impact on IL-2 production (Fig 3F), as well as a small, but reproducible impact on both CD4^+^ and CD8^+^ T cell proliferation (Fig 3G), achieving a maximal inhibition of around 30%. Mycophenolate mofetil, on the other hand, inhibited both IL-2 production (Fig 3H) and T cell proliferation (Fig 3I). Surprisingly, rapamycin did not have an impact on either IL-2 production or T cell proliferation. While the *in vitro* impact of rapamycin has been evaluated mostly in the context of purified T cells, it is possible that the responses we observed here might be driven by differences due to the tissue complexity in PBMCs. We did not evaluate the impact of rapamycin from purified T cells in the same donor. We also evaluated mycophenolate mofetil (MMF) and leflunomide, two compounds which generate the active metabolites mycophenolic acid and teriflunomide, respectively. While data from the published literature has largely described the impact of these compounds in purified T cell populations, our data here demonstrate that there is concordance (e.g., SYK/ZAP70, JAK and PI3Kδ) as well as some discrepancies (i.e., rapamycin) with the impact of these compounds on T cell activation and proliferation in a PBMC setting.

### The effect of small molecule inhibitors on reactive oxygen species (ROS) production by human phagocytes

**Fig 4:**
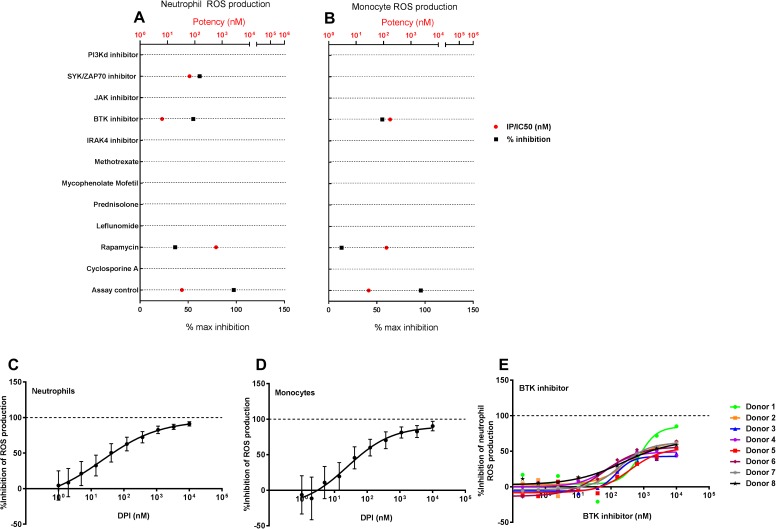
The effect of small molecule inhibitors on reactive oxygen species (ROS) production by human phagocytes. Ten minutes following stimulation with opsonized bacteria, the production of ROS in neutrophils and monocytes from human whole blood was evaluated using flow cytometry. Small molecule inhibitors inhibit ROS production neutrophils (A) and monocytes (B) in human whole blood following stimulation with opsonized bacteria. For each compound, the potency (red circles) and the percentage maximal inhibition achieved in the assay (black squares) are plotted along the X-axis. The potencies of the evaluated compounds in these assays are displayed as IC_50_ values. A maximum of 100% inhibition is possible for small molecule inhibitors in these assays. The reported potency and % max inhibition values were generated from a composite of 8–10 point dose response curves from n = 6–8 donors for each compound. The positive control used in this assay (Diphenyliodonium chloride [DPI], an NADPH oxidase inhibitor), can inhibit ROS production from neutrophils (C) or monocytes (D) in a dose dependent manner. A BTK inhibitor consistently inhibited ~50% neutrophil ROS production (E) across multiple donors evaluated in this assay. Each point in the dose response curve indicates mean±SEM of % inhibition at that dose from n = 6–8 donors.

Since phagocytes are at the forefront of pathogen clearance and immune defense, we evaluated the impact of SM inhibitors on reactive oxygen species (ROS) production by neutrophils (Fig 4A) and monocytes (Fig 4B) in human whole blood following incubation with opsonized bacteria. While diphenyliodonium chloride (an NADPH oxidase inhibitor) was able to completely inhibit ROS production from neutrophils (Fig 4C) and monocytes (Fig 4D), most of the evaluated SM inhibitors had minimal to no effect on neutrophil ROS production. Among the compounds tested, a SYK/ZAP70 inhibitor demonstrated a ~50% reduction in neutrophil ROS production in human blood. This is similar to previous reports of reduced respiratory burst in SYK deficient mouse neutrophils generated in bone marrow chimera studies [15]. Both the BTK inhibitors evaluated in the study led to a 30–50% reduction in neutrophil and monocyte ROS production (Fig 4A). Inhibition of neutrophil ROS production by a BTK inhibitor was very consistent across multiple donors evaluated in the study (Fig 4E). Rapamycin demonstrated a dose-dependent inhibition of neutrophil and monocyte ROS production, with a 20–40% maximal inhibition achieved in the assay. The other compounds evaluated in the study did not demonstrate inhibitory activity in the neutrophil ROS production assay.

### The effect of small molecule inhibitors on human natural killer (NK) cell mediated killing of target (K562) cells

**Fig 5:**
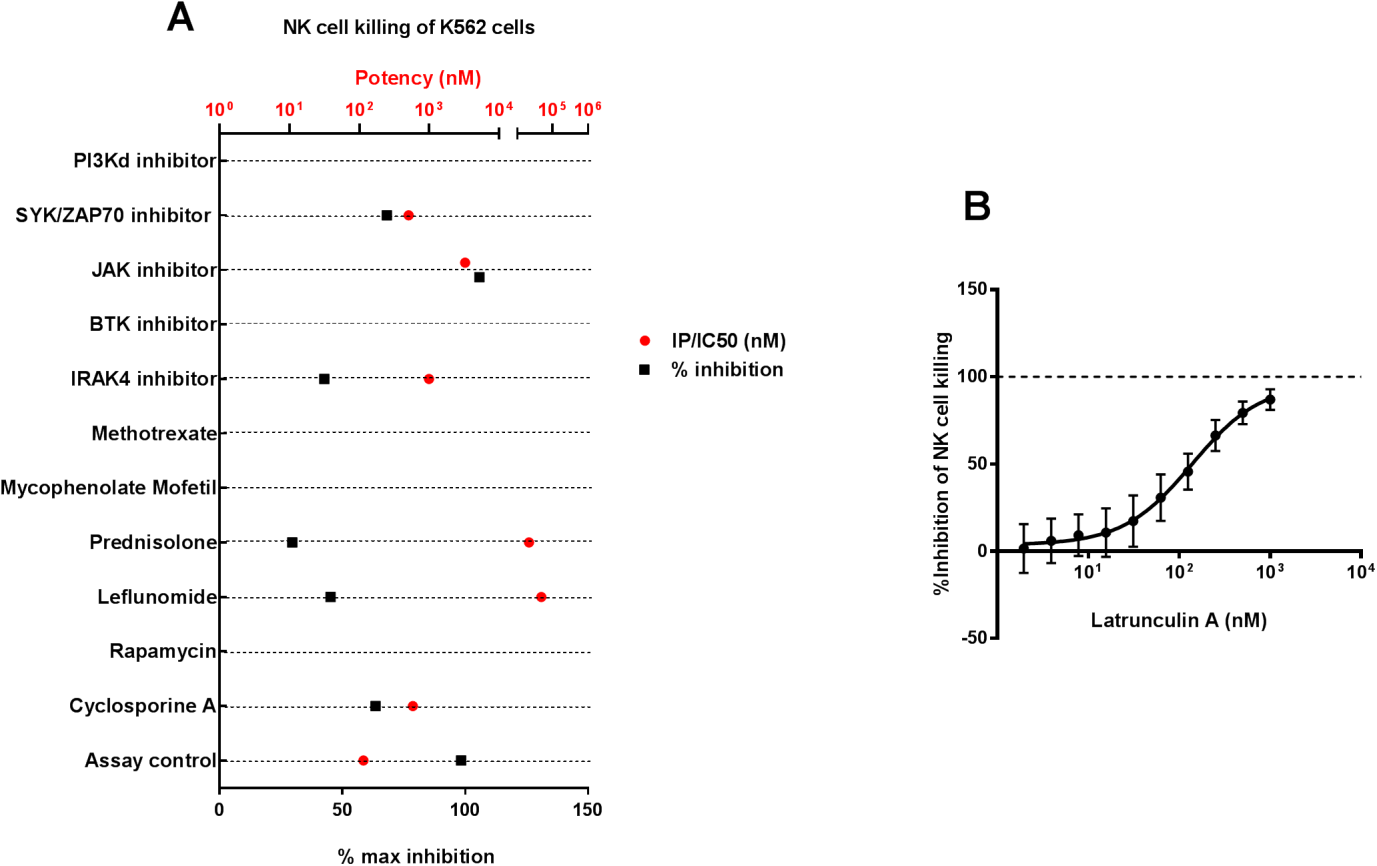
The effect of small molecule inhibitors on human Natural Killer (NK) cell mediated killing of target (K562) cells. The killing of MHC class I negative K562 cells (target cells) by NK cells in human PBMCs was evaluated by flow cytometry 2 hours following co-incubation at a ratio of 50:1 PBMC:target cells. Small molecules inhibit the killing activity of NK cells (A) in human PBMCs to varying degrees. For each compound, the potency (red circles) and the percentage maximal inhibition achieved in the assay (black squares) are plotted along the X-axis. The potencies of the evaluated compounds in these assays are displayed as IC_50_ values. A maximum of 100% inhibition is possible for small molecule inhibitors in these assays. The reported potency and % max inhibition values were generated from a composite of 8–10 point dose response curves from n = 6–8 donors for each compound. The positive control in this assay (Latrunculin A, an actin polymerization inhibitor), can inhibit NK cell killing (B) in a dose dependent manner. Each point in the dose response curve indicates mean±SEM of % inhibition at that dose from n = 7 donors.

Natural killer cells are critical in the clearance of virus infected cells and tumor cells that do not express MHC class I expression, thus evading CD8^+^ cytotoxic T cell mediated killing. We evaluated the impact of SM inhibitors on NK cell killing activity in a co-culture assay containing PBMCs as effector cells (that contain NK cells in the PBMC population) and MHC class I negative K562 cells as target cells (Fig 5A). A varying degree of maximal inhibition of NK cell killing activity was observed with the SM inhibitors. Among the SM inhibitors evaluated, a SYK/ZAP70 inhibitor had a discernible dose-dependent inhibition of NK cell killing activity, although only ~50% of the response was inhibited even with the highest concentrations tested. On the contrary, the killing activity of NK cells in this assay was completely inhibited by the actin polymerization inhibitor Latrunculin A (Fig 5B).

### The effect of small molecule inhibitors on TLR induced cytokine/chemokine production in human PBMCs

**Fig 6:**
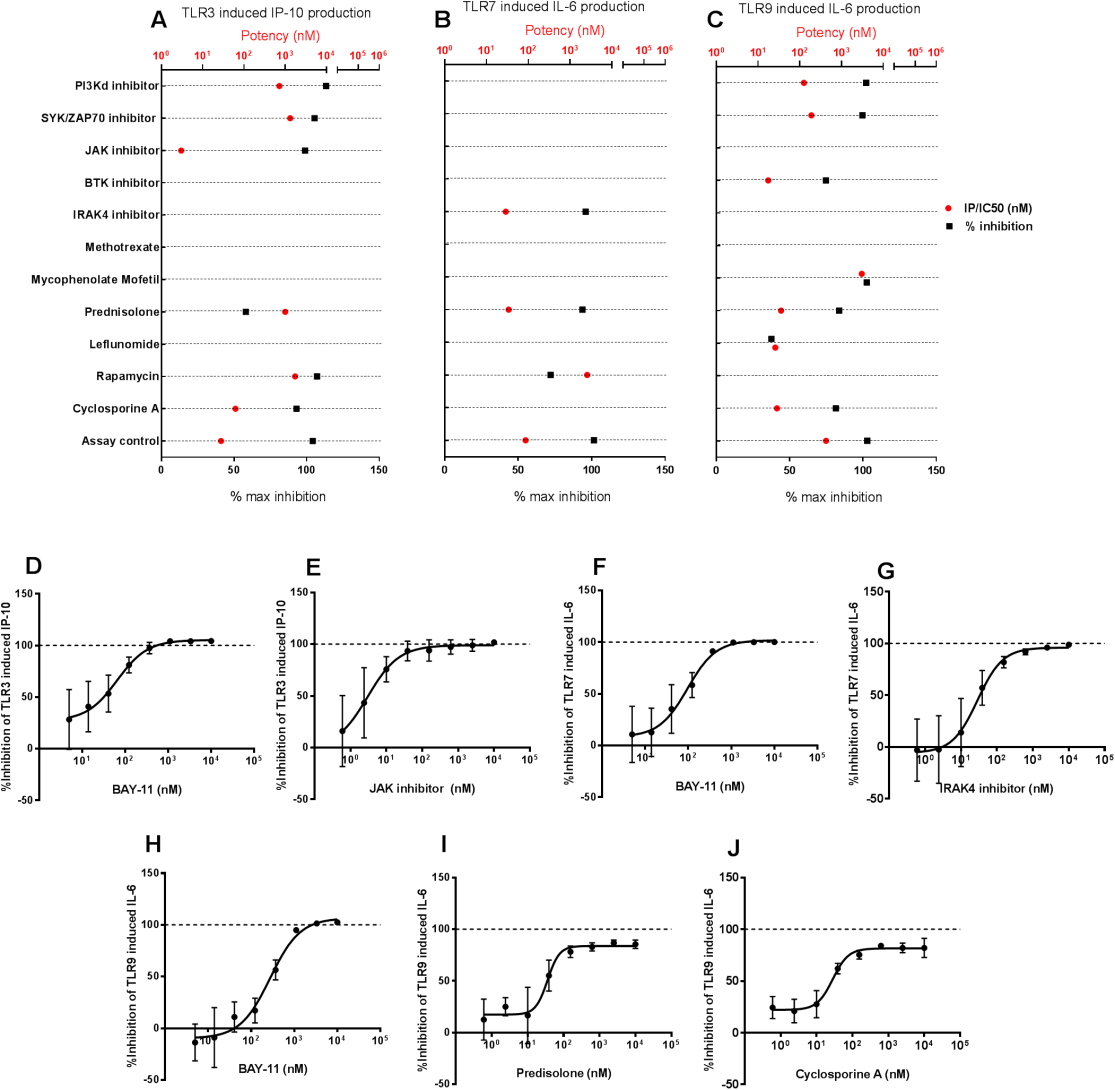
The effect of small molecule inhibitors on TLR induced cytokine responses in human PBMCs. Cytokine production in PBMC culture supernatants 24h following stimulation with TLR agonist ligands were evaluated. Small molecule inhibitors suppress TLR3 agonist induced IP-10 production (A), TLR7 agonist induced IL-6 production (B) and TLR9 agonist induced IL-6 production (C) in human PBMCs *in vitro*. For each compound, the potency (red circles) and the percentage maximal inhibition achieved in the assay (black squares) are plotted along the X-axis. The potencies of the evaluated compounds in these assays are displayed as IC_50_ values. A maximum of 100% inhibition is possible for small molecule inhibitors in these assays. The reported potency and % max inhibition values were generated from a composite of 8–10 point dose response curves from n = 6 donors for each compound. Dose-dependent inhibition of TLR responses by a positive control (BAY-11) or small molecule inhibitors (D–J). Each point in the dose response curve indicates mean±SEM of % inhibition at that dose from n = 6 donors.

Toll-like receptors are key mediators in the recognition of pathogen associated molecular patterns that trigger innate immune responses via production of pro-inflammatory cytokines and chemokines. We evaluated the impact of SM inhibitors on the production of cytokines and chemokines from human PBMCs upon stimulation of three key TLR ligands. Production of IFNγ-inducible protein 10 (IP-10) was used as a read-out of TLR3 stimulation in PBMCs with high molecular weight Poly (I:C). While the small molecule inhibitors inhibited the Poly (I:C) induced IP-10 response to varying degrees (Fig 6A), the positive control BAY-11 fully inhibited the response (Fig 6D). Among the SM inhibitors evaluated, a JAK inhibitor demonstrated a dose-dependent inhibition profiles with near maximal inhibition of the response (Fig 6E). Production of IL-6 was used as a read-out of TLR7 stimulation in PBMCs with the synthetic TLR7 specific agonist CL307. While the small molecule inhibitors inhibited this response to varying degrees (Fig 6B), the positive control BAY-11 fully inhibited this response (Fig 6F). As expected, an IRAK4 inhibitor demonstrated the best dose-dependent inhibition profiles, with near maximal inhibition of CL307 induced IL-6 production in this assay (Fig 6G). Production of IL-6 was used as a read-out of TLR9 stimulation in PBMCs with the synthetic TLR9 agonist ODN 2395. While the small molecule inhibitors inhibited the CL307 induced IL-6 production to varying degrees (Fig 6C), the positive control BAY-11 fully inhibited this response (Fig 6H). Similar to the PI3Kδ inhibitor and the SYK/ZAP70 inhibitor, prednisolone (Fig 6I) and cyclosporine (Fig 6J) also demonstrated a dose-dependent inhibition of TLR9 response in this assay.

### Evaluating exposure-function relationship of small molecule inhibitors

**Fig 7:**
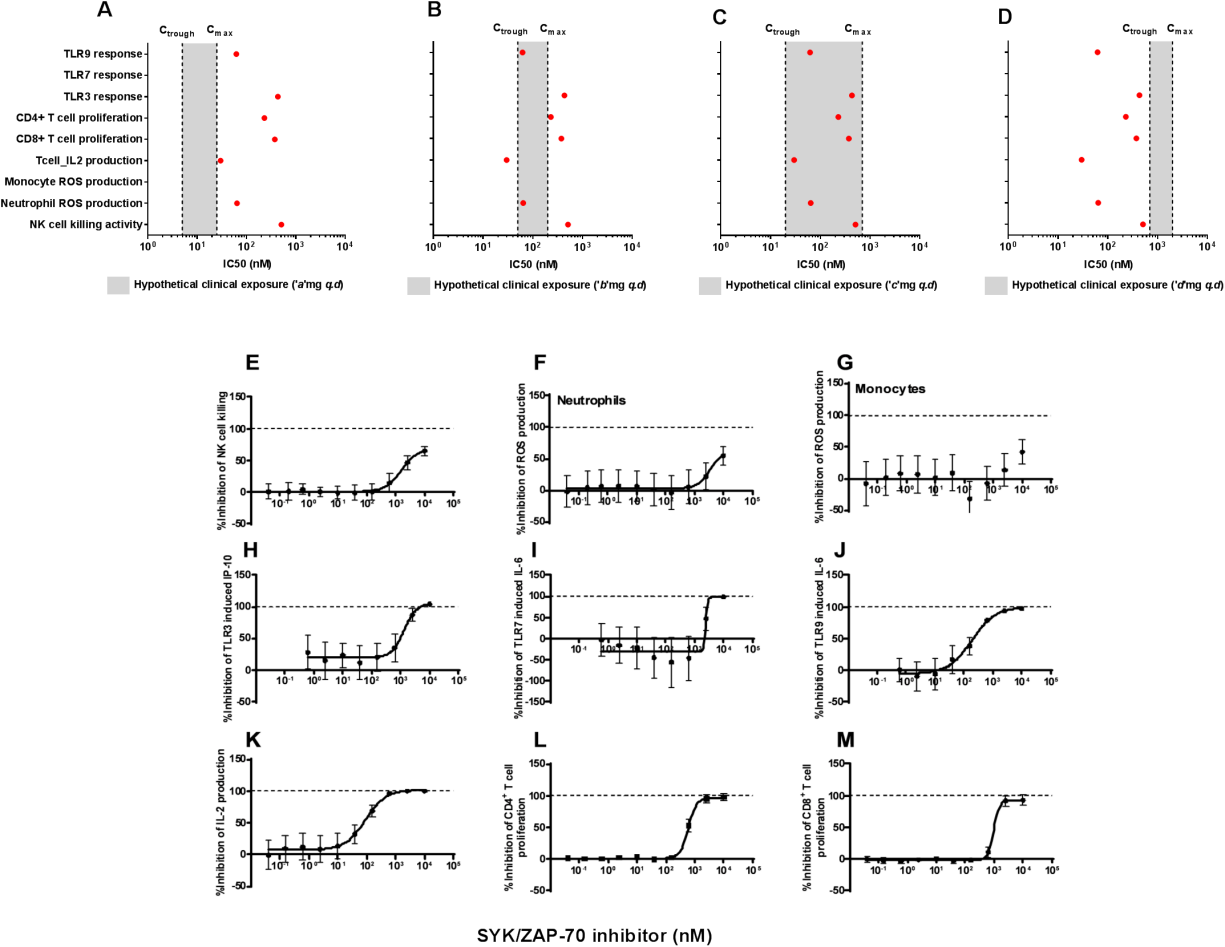
Evaluating clinical relevance of the immune function impact of a SYK/ZAP-70 inhibitor. (A–D) Relationship between the *in vitro* immune function impact and hypothetical clinical exposure levels of a SYK/ZAP-70 inhibitor. The potency (red circles) of SYK/ZAP-70 inhibitor in the individual assays are overlaid on the hypothetical clinical exposure levels of SYK/ZAP-70 inhibitor (grey box). The potencies of the evaluated compounds in these assays are displayed as an IC_50_ in the dose response experiments. A maximum of 100% inhibition is possible for small molecule inhibitors in these assays. The reported potency and % max inhibition values were generated from a composite of 8–10 point dose response curves from n = 6–8 donors for each compound. The immune function impact of the SYK/ZAP-70 inhibitor on NK cell killing (E), neutrophil ROS production (F), monocyte ROS production (G), response to TLR3 agonist (H), response to TLR7 agonist (I), response to TLR9 agonist (J), T cell IL-2 production (K), CD4^+^ T cell proliferation (L) and CD8^+^ T cell proliferation (M) are depicted as mean±SEM of % inhibition of that response from n = 6–8 donors for each assay. The Neutrophil and monocyte phagocyte responses were evaluated in human whole blood, while all the other responses were evaluated in PBMCs. The potencies depicted in A are corrected for plasma protein binding, using the potencies obtained from dose-response experiments depicted in E-M.

The plasma concentration of a compound in a given dosing paradigm is a key factor that determines the immune function impact of the compound. To evaluate if the immune function impact profiles we have generated *in vitro* would have value in predicting clinical outcome, we evaluated the relationship of the *in vitro* immune function potencies of the SYK/ZAP-70 inhibitor to hypothetical clinical exposures, depicted as a range of maximal (C_max_) and minimal (C_trough_) plasma concentrations in a hypothetical dosing paradigm (Fig 7A–7D). The dose-response curves for the immune functions evaluated are depicted in Fig 7E–7M. While the dose-response curves were generated with the SYK/ZAP-70 inhibitor in our assay platform, the clinical exposures were hypothetical values used to evaluate the immune function impact of the SYK/ZAP-70 inhibitor. This exercise suggests that the IC_50_ values of certain immune functions could be in between or on either side of the C_trough_ and C_max_ plasma drug concentrations. At the hypothetical clinical exposures achieved by the SYK/ZAP-70 inhibitor at ‘*a*’ mg in a q.d dosing paradigm, the IC_50_ values of all the immune functions tested were above the hypothetical clinical C_max_, suggesting that the immune functions evaluated may not be impacted at the IC_50_ levels (Fig 7A). On the other hand, at the hypothetical clinical exposures achieved by the SYK/ZAP-70 inhibitor at ‘*b*’ mg in a q.d dosing paradigm, the T cell IL-2 production will always be below the clinical C_trough_, suggesting that at least 50% of IL-2 production by T cells will always be impacted by this compound at this dosing paradigm (Fig 7B). In hypothetical clinical exposures achieved by the SYK/ZAP-70 inhibitor at ‘*c*’ mg in a q.d dosing paradigm, all the immune functions are within the clinical exposure range, suggesting that these functions will be impacted to a certain extent (Fig 7C). Finally, in hypothetical clinical exposures achieved by the SYK/ZAP-70 inhibitor at ‘*d*’ mg in a q.d dosing paradigm, all the immune functions are below the clinical C_trough_, suggesting that all these immune functions will be inhibited at least by 50% during the dosing paradigm (Fig 7D). If projected human exposures are available for a given compound in the preclinical discovery stage, similar *in vitro* potency–exposure relationship evaluations could be performed to assess the immune function impact of compounds in the context of benefit-risk assessment.

### Gene expression profiles help distinguish the in vitro impact of small molecule inhibitors in the T cell stimulation assay

**Fig 8:**
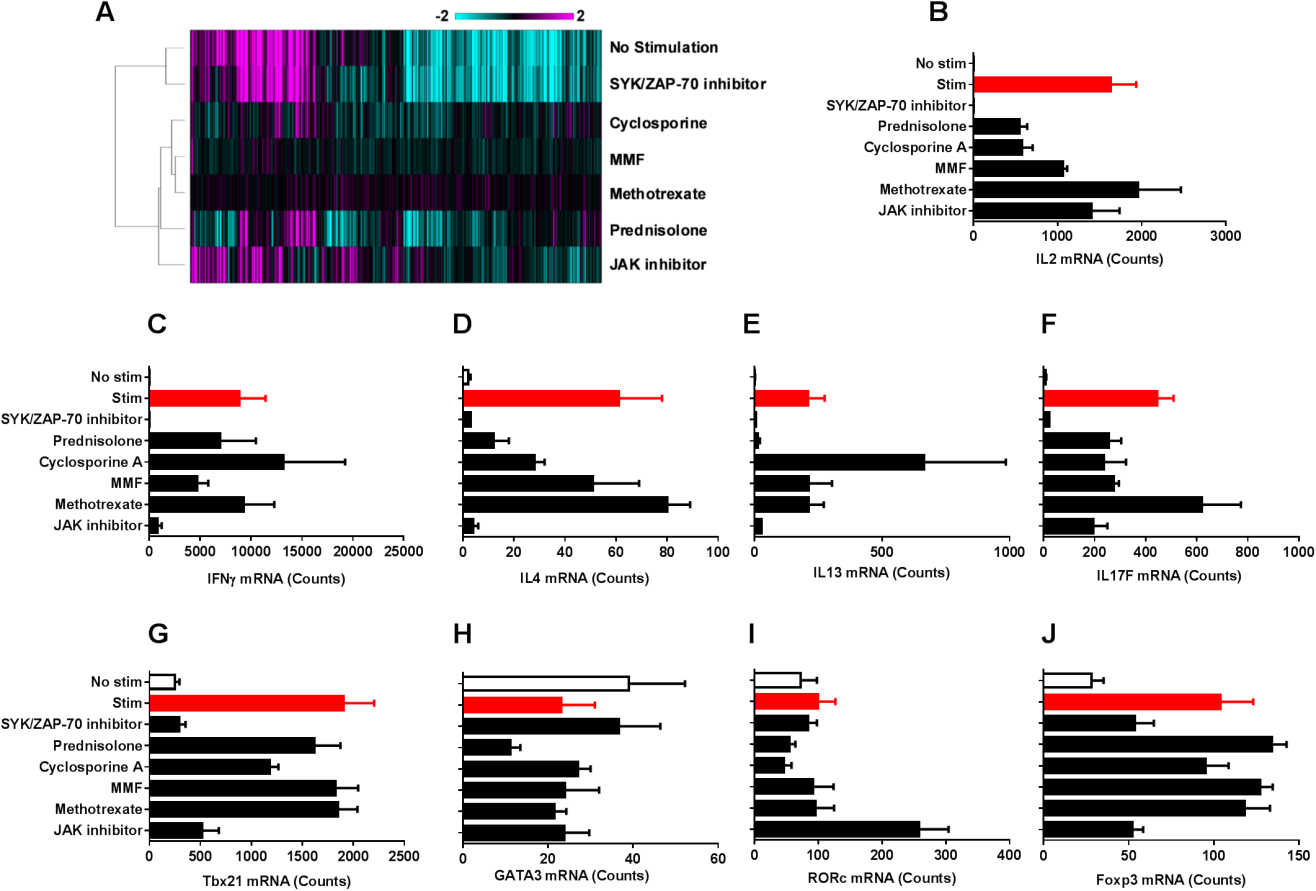
Impact of small molecule inhibitors on gene expression profiles in the T cell stimulation assay. (A) A nanostring gene expression panel was used to evaluate mRNA profiles of PBMCs 24 h following treatment with 1μM of the SM inhibitors in the T cell stimulation assay. The gene expression profiles of unstimulated and compound treated stimulated samples are shown. All data were normalized to housekeeping genes and stimulated DMSO control samples. Hierarchical agglomerative clustering of genes with greater than a 2-fold change (p-value<0.05) is shown. (B) Transcript expression of IL-2 under unstimulated, stimulated, and compound treated conditions. The mRNA expression levels of the cytokine genes IFNγ (C), IL4 (D), IL13 (E), IL17F (F) as well as the transcription factors Tbx21 (G), GATA3 (H), RORc (I) and Foxp3 (J) are depicted as examples from the gene expression dataset. The gene expression profiles in (A) is a composite of PBMCs from n = 3 donors, while the individual gene expression profiles in (B-J) are mean±SEM of mRNA expression from n = 3 PBMC donors.

**Fig 9:**
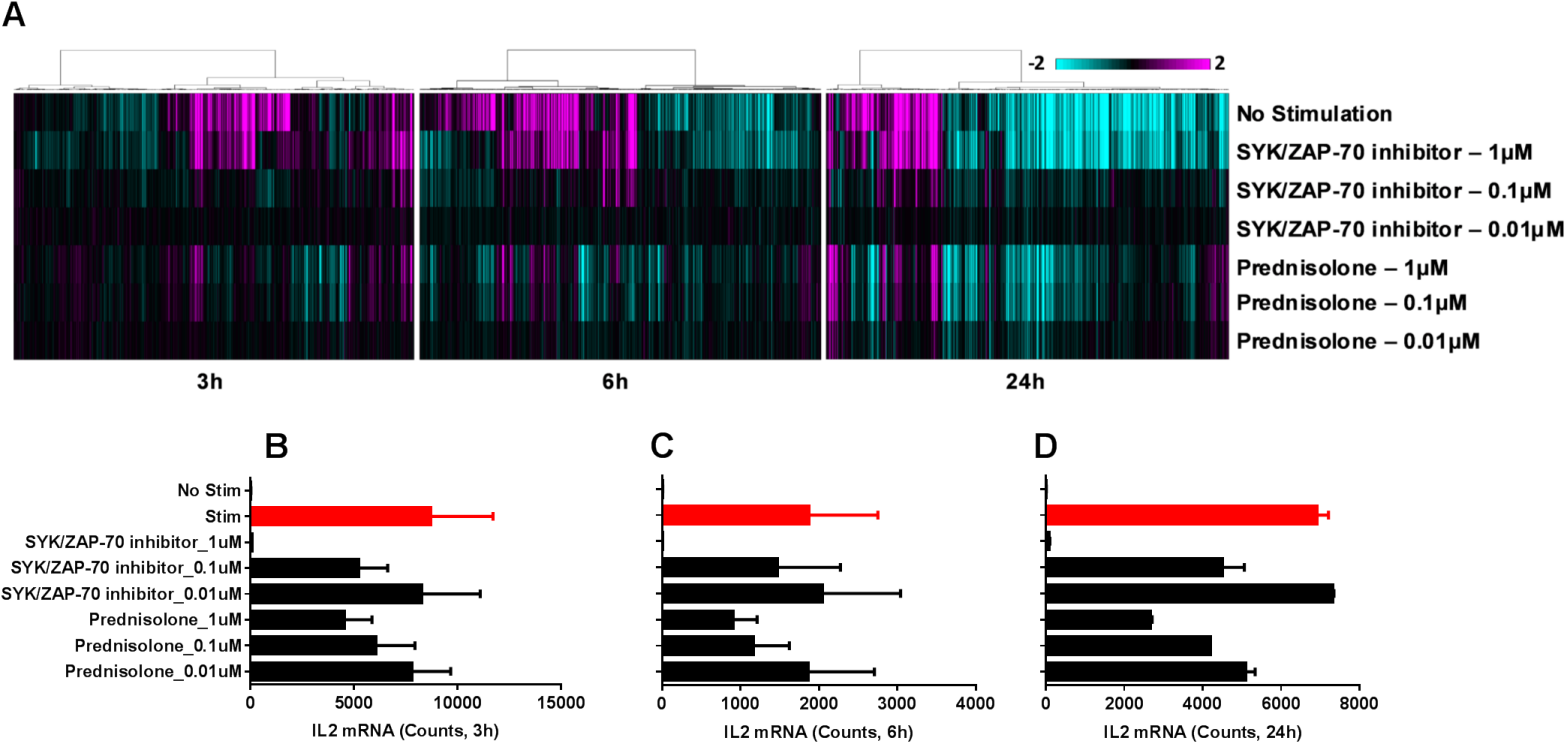
Impact of SYK/ZAP-70 inhibitor and prednisolone on gene expression profiles in the T cell stimulation assay. (A) A nanostring gene expression panel was used to evaluate mRNA profiles of PBMCs 3, 6 and 24 h following treatment with three different concentrations of SYK/ZAP-70 inhibitor and prednisolone. The gene expression profiles of unstimulated and compound treated stimulated samples are shown. All data were normalized to housekeeping genes and stimulated DMSO control samples. Hierarchical agglomerative clustering of genes with greater than a 2-fold change (p-value<0.05) is shown. Transcript expression of IL-2 under unstimulated, stimulated, and stimulated plus SYK/ZAP-70 inhibitor or prednisolone treated conditions at the 3h (B), 6h (C) and 24h (D) time points in the T cell stimulation assay. The gene expression profiles in (A) is a composite of PBMCs from n = 3 donors, while the individual gene expression profiles in (B-D) are mean±SEM of mRNA expression from n = 3 PBMC donors.

Since the functional impact of small molecule inhibitors described in the previous assays were evaluated based on a single read-out (i.e., cytokine production, proliferation, killing function), we sought to evaluate the gene expression profiles of a subset of these small molecule inhibitors in the T cell stimulation assays to gain insight into the broader impact of these inhibitors. We evaluated the impact of a single concentration (1μM) of seven small molecule inhibitors with different MoAs 24 h following stimulation of human PBMCs with anti-CD3 and anti-CD28 using a nanostring gene expression kit. Our data demonstrate that among the compounds evaluated, the SYK/ZAP-70 inhibitor fully inhibited T-cell activation, and the gene expression profile of SYK/ZAP-70 inhibitor was very similar to the gene expression profile of unstimulated PBMC controls (Fig 8A). Cyclosporine, MMF and methotrexate demonstrated minimal impact on restoring the gene expression profile to the level of unstimulated PBMC samples (Fig 8A). Prednisolone and JAK inhibitors modified the gene expression profile of stimulated samples similar to that of unstimulated cells, albeit not to the extent of SYK/ZAP-70 inhibitor (Fig 8A). Under these stimulation conditions, the mRNA expression of IL-2, IFNγ, IL-4, IL-13 and IL-17F were upregulated, and SYK/ZAP-70 inhibitor suppressed the expression of all these cytokines (Fig 8B–8F). Among the key transcription factors evaluated, Tbx21 (which encodes T-bet) and Foxp3 were upregulated under these stimulation conditions, and SYK/ZAP-70 inhibitor inhibited this response (Fig 8G–8J). The JAK inhibitor evaluated in this study did not reduce the expression of IL-2 (Fig 8B), however it reduced the expression of IFNγ, IL-4 and IL-13 under these stimulation conditions (Fig 8C–8E). Interestingly, prednisolone reduced the expression of IL-4 (Fig 8D) and IL-13 (Fig 8E) but not IFNγ (Fig 8C). Furthermore, a time- and dose-dependent change in the transcriptomics profile was observed in this assay upon treatment with SYK/ZAP-70 inhibitor or prednisolone (Fig 9A). As an example, the mRNA expression of IL-2 was impacted in a time- and dose-dependent manner by SYK/ZAP-70 inhibitor and prednisolone at 3h (Fig 9B), 6h (Fig 9C) and 24h (Fig 9D) following stimulation.

## Discussion

The compounds that antagonize the function of drug targets in the immune system straddle a thin line between efficacy and nonspecific immunosuppression, and the systematic evaluation of the qualitative and quantitative impact of these compounds on the immune system is rather limited. Since the immune system and its manifestations are multifaceted, contextual and pleiotropic in terms of impact, a high-dimensional, non-reductionist, systems immunology approach has been proposed to better understand the system-wide impact of key cellular and molecular mediators in the immune system [16–18]. Along similar lines, a systems pharmacology approach to better understand the impact of compounds and prediction of efficacy and safety have also been proposed, a theme that is also gaining interest with regulatory agencies [19–22]. Using a human tissue-based immune function assay platform, here we demonstrate that the evaluation of a comprehensive ‘immune function impact’ of pharmacological modulators may help better understand the benefit-risk profiles of compounds that target the immune system.

Since an immune outcome depends on the concerted interaction of multiple immune cells, we decided to retain the tissue complexity intact and established the cell-based functional assays in human PBMCs or whole blood, rather than using isolated cell populations. Here we demonstrate that such a streamlined use of immune function assays can be used to generate a qualitative and quantitative assessment of pharmacological inhibitors in the context of their impact on the immune system. While similar platforms have been used in the past to evaluate the broader functional profiles of compounds using co-culture of isolated cell populations [23–25], our platform has focused on employing endogenous primary human tissues to evaluate the immune system impact of pharmacological modulators.

While confirming the MoA-associated functions of these small molecule inhibitors, we also observed some discrepancies. For example, the PI3Kδ inhibitor evaluated reduced IL-2 production, but not CD4^+^ and CD8^+^ T cell proliferation in a PBMC-based T cell activation assay. These results are consistent with previous reports that demonstrate that CD28 costimulation reduces the impact of PI3Kδ inhibitors in a PBMC-based setting, leading to unimpaired T cell proliferation [26]. On the other hand, a SYK/ZAP-70 inhibitor evaluated inhibited IL-2 production as well as CD4^+^ CD8^+^ T cell proliferation in the same assay. Interestingly, we also found that a BTK inhibitor inhibited IL-2 production, but had limited impact on CD4^+^ and CD8^+^ T cell proliferation. It is possible that this could be due to off-target activity of this compound on ITK in T cells in this cell-based assay system, along the lines of previous reports that demonstrate that the clinically approved irreversible BTK inhibitor ibrutinib also irreversibly binds to ITK and impacts T cell function [27]. The effect of JAK inhibitors on IL-2 production could be due to their impact on cytokine-induced positive feedback rather than a direct impact of IL-2 production driven by the proximal signaling cascade upon TCR activation [28]. While the long-term responses that shape immunological memory cannot be optimally evaluated in this 72 hour assay, longitudinal profiling of clinical PBMC samples from compound-treated patients might provide additional insights into the *in vivo* impact of these compounds in shaping immunological memory.

Similarly, we were also able to generate qualitative and quantitative immune impact profiles of small molecule inhibitors on the innate immune system. While we observed inhibition of neutrophil ROS production by small molecule inhibitors of BTK, a previous report has demonstrated that neutrophils from XLA patients who lack BTK demonstrate increased ROS production upon stimulation with PMA and TLR ligands [29]. It is possible that the discrepancy in these findings may be either due to the stimuli used or due to the differences between pharmacological antagonism and genetic deficiency or donor-dependent variations due to genetic/environmental factors. Genetic deficiency in signaling molecules may sometimes lead to upregulation of compensatory mechanisms, such as the increased cytokine production observed in BTK deficient cells of monocytic lineage [30]. Among the inhibitors evaluated, a SYK/ZAP-70 inhibitor demonstrated a consistent inhibition of NK cell killing activity. This data is consistent with previous reports demonstrating that Syk kinase is essential for mediating NK cell killing function [31]. While a majority of the TLR signaling pathways are mediated by IRAK4 kinase [32], we observed that inhibitors of other kinases such as PI3Kδ, SYK/ZAP-70 and JAK also have an impact on functional TLR responses, consistent with previous reports [33–35]. Among the TLR responses evaluated, cyclosporine, PI3Kδ and SYK/ZAP70 inhibitors inhibited TLR3 and TLR9, but not TLR7 induced responses. On the other hand, JAK inhibitors achieved maximal inhibition of TLR3 responses while inhibition of TLR7 and TLR9 responses were either partial or did not generate a sigmoidal dose-response. The IRAK4 inhibitors were the most potent in inhibiting TLR7 responses, while cyclosporine and prednisolone were potent in inhibiting TLR9 responses.

While this assay platform was established in a one assay–one readout format to evaluating the impact of compounds, largely to facilitate a potency-based qualitative comparison, the impact of these compounds are broader in the immune system. Transcriptomics profiling of a subset of these compounds at a single concentration (1μM) in the T cell IL-2 production assay demonstrated that SYK/ZAP-70 inhibitor reverted the gene expression profile of activated T cells to that of unstimulated T cells, while cyclosporine, MMF and methotrexate had limited impact. Prednisolone and JAK inhibitors modified the gene expression profiles of activated T cells, but not to the extent of SYK/ZAP-70 inhibitor. While prednisolone, cyclosporine and MMF partially reduced IL-2 mRNA levels, SYK/ZAP-70 inhibitor almost completely inhibited IL-2 mRNA expression in this assay.

To determine if we can use the *in vitro* potencies obtained from this assay platform to evaluate or project clinical outcomes, we used hypothetical clinical exposures of a SYK/ZAP-70 inhibitor to evaluate immune function impact. We found that while the SYK/ZAP-70 inhibitors impact TLR responses, T cell responses and NK cell killing, the clinical exposures of the compound will ultimately define the nature and the outcome of the immune function impact. For example, in the hypothetical exposure-function relationships we have explored in Fig 7A–7D, if the IC_50_ of the compound for an immune function was below C_min_, impacting immune function at that level would depend on role of that immune function in driving efficacy, safety or both. For example if it was an efficacy-driving immune function that does not play a major role in the control of certain infections, it is possible to speculate that inhibition of that function at that clinical exposure might have a reduced probability of leading to infection-related adverse events. While these could be predictive to a certain extent, it may not be possible to fully elucidate with certainty the real-world clinical impact of the compound. However, a rational evaluation of such relationships during the preclinical drug discovery with projected human exposures might help better evaluate the larger immune function impact of compounds rather than speculation of mechanistic impact. While we have evaluated such a relationship in the context of IC_50_ values, the same exercise could be done with any level of inhibition (i.e., IC_20_, IC_80_) as necessary, for a given compound.

**Fig 10:**
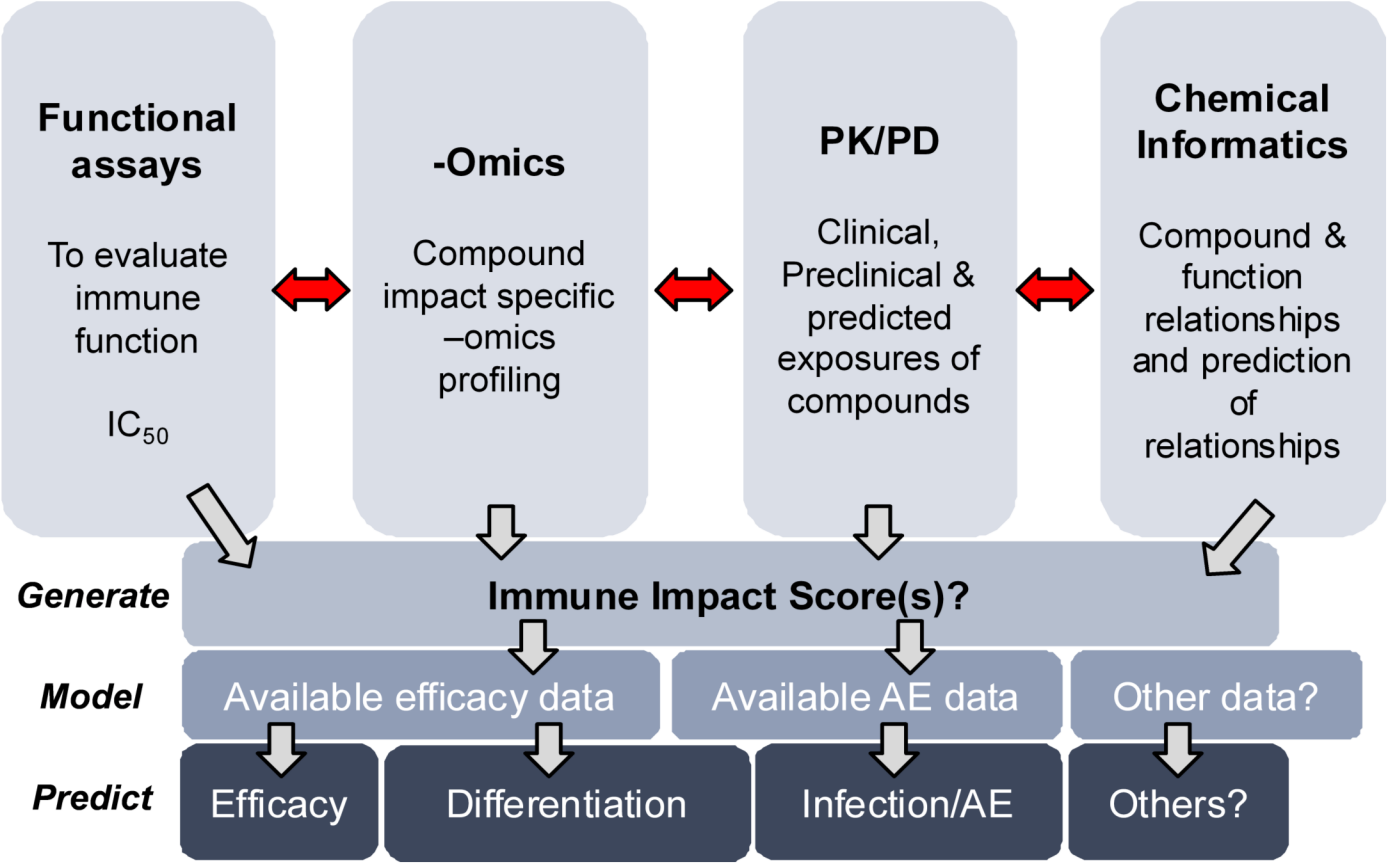
A conceptual framework to develop an ‘immune impact score’ for compounds. The impact of compounds in immune system-based functional assays, -omics approaches, PK/PD information and chemical informatics (that classify and predict compound-function relationships based on available information) might be integrated with the aim of assigning an ‘immune impact score’ for individual compounds. Further integration of available or modeled efficacy and adverse event data for compounds with the immune impact score might be useful in better understanding and predicting the efficacy, adverse event profile and differentiation of these compounds in a clinical setting. PK/PD–Pharmacokinetics/Pharmacodynamics, AE–adverse events.

While the on-target and off-target activities of small molecule inhibitors are concentration-dependent *in vitro* and *in vivo*, we used this assay platform as a mechanism-agnostic screening system to evaluate the larger ‘immune system impact’ of small molecule inhibitors against immune system targets. The efficacy, safety and adverse event profile of a compound in a clinical setting can be more heterogeneous and different compared to its impact observed in the non-clinical stages of the drug discovery process. However, it may be possible to integrate data from compound-based functional assays, transcriptomics, genomics, clinical or projected exposures and chemical informatics to generate an ‘immune impact score’ for a given compound (Fig 10). If efficacy and adverse event profile data are available for a compound or a class of compounds that share the MoA, it is possible to integrate these data and the immune impact score to model or predict clinical benefit-risk profiles.

A streamlined use of such immune function assay platforms might enable a more consistent evaluation of pharmacological modulators in the context of immune system impact. Such platforms, data and interpretations have limitations, since they cannot be exhaustive, encompassing and fully predictive, and have to be constantly updated based on emerging biology. For example, although these studies were performed using circulating immune cells from healthy human donors, the response profiles for the same pharmacological inhibitors may be different in tissue-resident immune cells or immune cells from diseased patients. Although we have not incorporated selectivity profiles of the inhibitors in our analyses, it is possible that integration of such datasets into these analyses might modify the interpretations of these findings. Furthermore, since new therapies for chronic inflammatory or autoimmune disorders are evaluated and used in combination with currently available therapies, it is also possible to evaluate the ‘combination immune impact’ using such platforms. Such integrative immunopharmacology approaches might be helpful for the successful translation of non-clinical drug discovery to clinical success in the future.

## Supporting information

S1 Fig Description of the parameters used in the figures. (A) In our assays and results, the IC_50_ describes the concentration of the compound that achieves half maximal inhibition of the activity in a sigmoidal dose-response curve. The % maximal inhibition is the maximal percentage of suppressive activity a compound can have in an assay, and not the concentration of the compound that gives the maximal suppression. (B) Sample profile of a SYK/ZAP-70 inhibitor in terms of its potencies and percentage of maximal inhibition in each of the immune function assays.
(TIF)

S2 Fig Flow cytometry gating strategies used in the assays. (A) T cell proliferation assay. Cells were gated on FSC/SSC properties > CD3 > CD4/CD8 > EdU to report the percentage of CD3^+^CD4^+^EdU^+^ or CD3^+^CD8^+^EdU^+^ cells used for analysis. (B) NK cell killing assay. Target (K562) cells were gated on FSC/SSC properties > GFP > Propidium iodide to report the percentage of GFP^+^PI^+^ cells used for analysis. (C) Phagocyte burst assay. Cells were gated on FSC/SSC properties > High content DNA > Neutrophil/monocyte subsets > ROS production. Geometric mean fluorescence intensities (GMFI) of ROS detection dye in neutrophils or monocytes were used for analysis.
(TIFF)

S1 Table Summary of assay formats, IC_50_ values and maximal responses of inhibitors in assays. IC_50_ values are corrected for plasma protein binding, except for assay controls in some of the assays.
(XLSX)
