# Supplementary figures and images for: A human tissue-based functional assay platform to evaluate the immune function impact of small molecule inhibitors that target the immune system
Source: PLoS One. 2017 Jul 18;12(7):e0180870. doi: 10.1371/journal.pone.0180870 (PMC5515432; doi:10.1371/journal.pone.0180870)

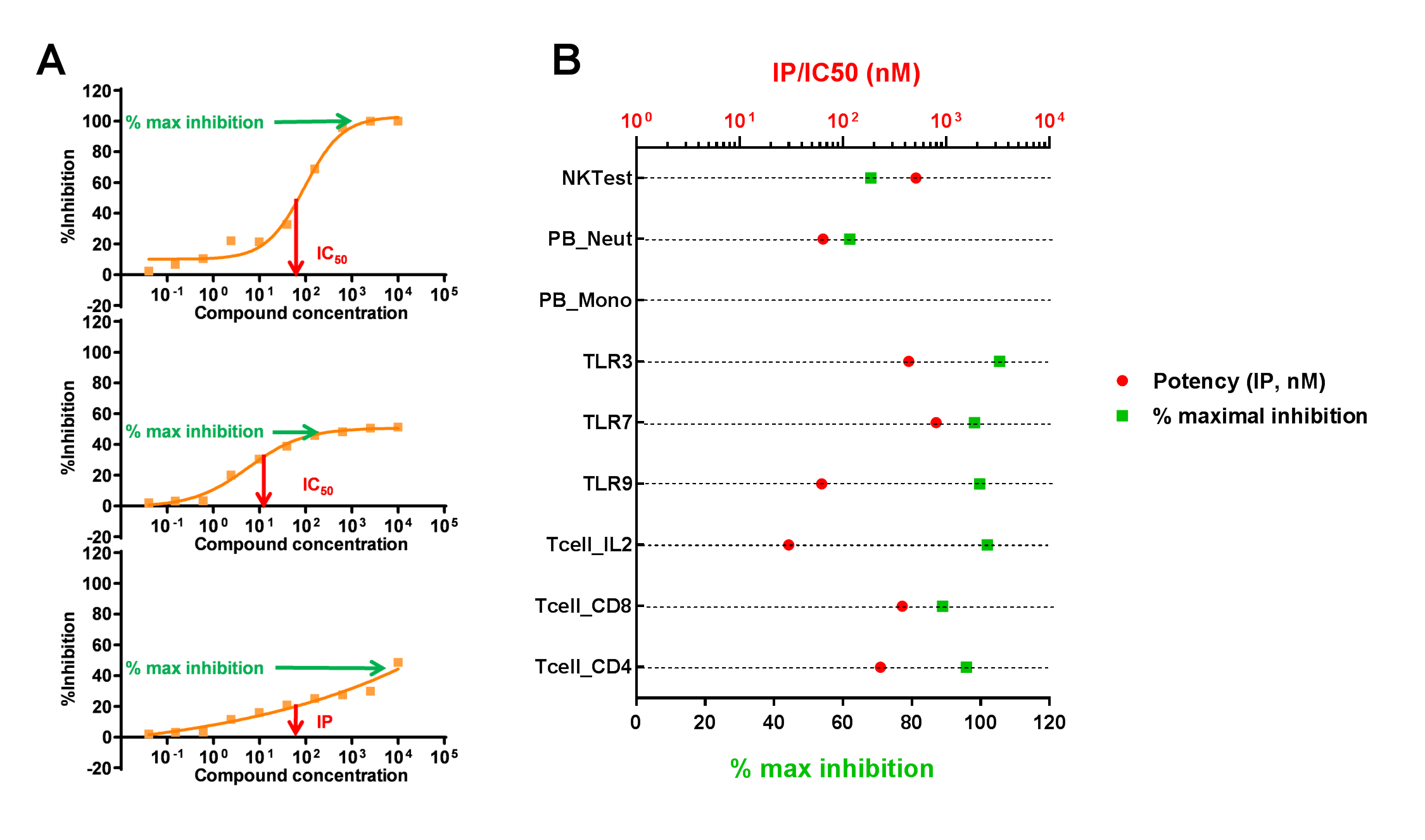

Supplement: S1 Fig — (A) In our assays and results, the IC50 describes the concentration of the compound that achieves half maximal inhibition of the activity in a sigmoidal dose-response curve. The % maximal inhibition is the maximal percentage of suppressive activity a compound can have in an assay, and not the concentration of the compound that gives the maximal suppression. (B) Sample profile of a SYK/ZAP-70 inhibitor in terms of its potencies and percentage of maximal inhibition in each of the immune function assays. (TIF) [file pone.0180870.s001.tif]

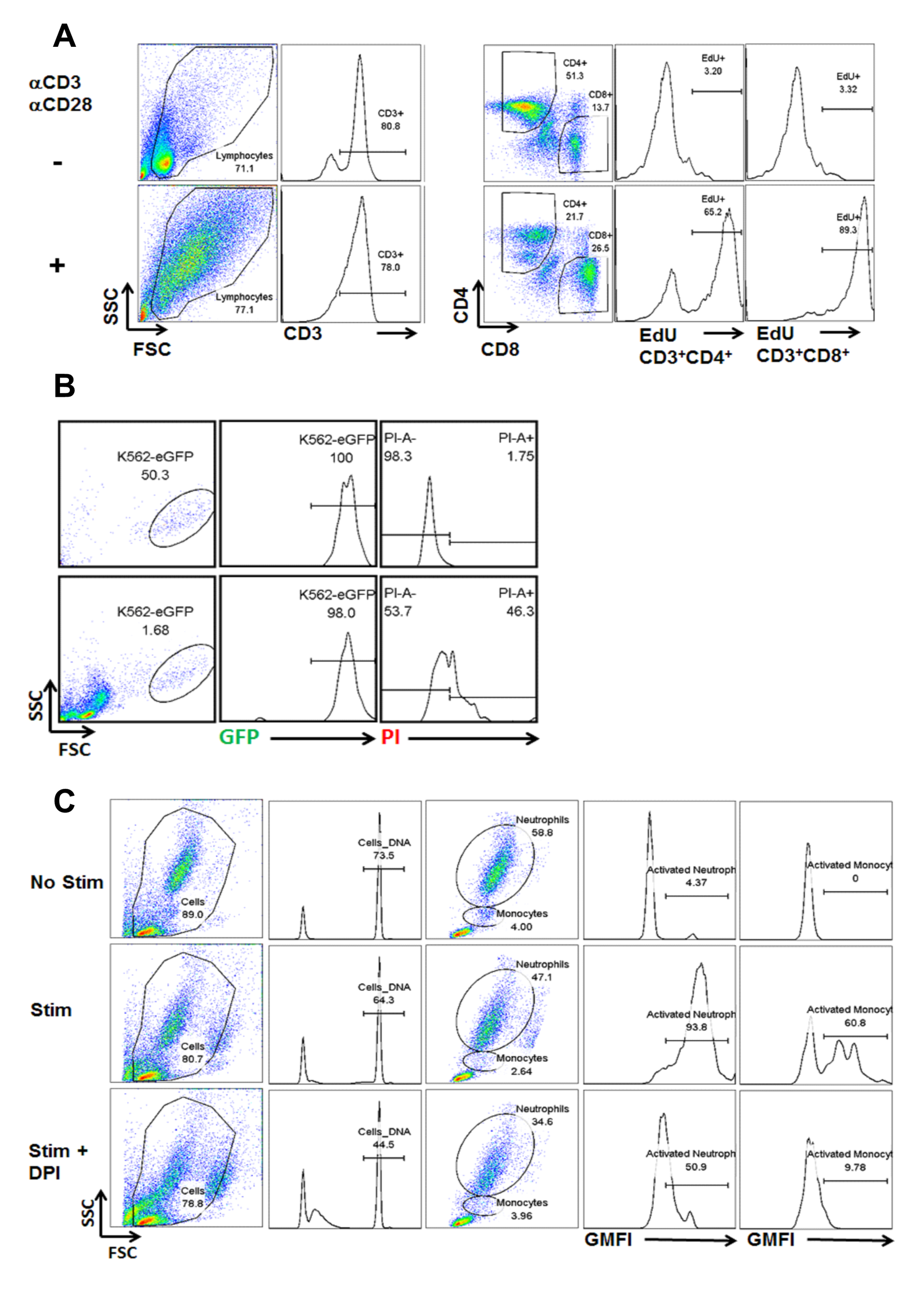

Supplement: S2 Fig — (A) T cell proliferation assay. Cells were gated on FSC/SSC properties > CD3 > CD4/CD8 > EdU to report the percentage of CD3+CD4+EdU+ or CD3+CD8+EdU+ cells used for analysis. (B) NK cell killing assay. Target (K562) cells were gated on FSC/SSC properties > GFP > Propidium iodide to report the percentage of GFP+PI+ cells used for analysis. (C) Phagocyte burst assay. Cells were gated on FSC/SSC properties > High content DNA > Neutrophil/monocyte subsets > ROS production. Geometric mean fluorescence intensities (GMFI) of ROS detection dye in neutrophils or monocytes were used for analysis. (TIFF) [file pone.0180870.s002.tiff]
